# Pretreatment ^18^ F-FDG uptake heterogeneity can predict survival in patients with locally advanced nasopharyngeal carcinoma——a retrospective study

**DOI:** 10.1186/s13014-014-0268-5

**Published:** 2015-01-08

**Authors:** Zhongyi Yang, Qi Shi, Yongping Zhang, Herong Pan, Zhifeng Yao, Silong Hu, Wei Shi, Beiling Zhu, Yingjian Zhang, Chaosu Hu

**Affiliations:** Department of Nuclear Medicine, Fudan University Shanghai Cancer Center, Shanghai, China; Department of Radiation Oncology, Fudan University Shanghai Cancer Center, Shanghai, China; Center for Biomedical imaging, Fudan University, Shanghai, China; Department of Oncology, Shanghai Medical College, Fudan University, No.270, Dong’an Road, Xuhui District, Shanghai, China

**Keywords:** Nasopharyngeal carcinoma, ^18^ F-FDG PET/CT, Prognosis, Heterogeneity

## Abstract

**Background:**

Intratumoural heterogeneity has been demonstrated to be a strong indicator of malignant transformation. Our study was to investigate pretreatment ^18^ F-FDG parameters, including ^18^ F-FDG based heterogeneity for predicting survival in patients with locally advanced nasopharyngeal carcinoma (NPC).

**Methods:**

Forty newly diagnosed, biopsy-proven locally advanced NPC patients who underwent ^18^ F-FDG PET/CT were retrospectively included. The following PET parameters were assessed: maximum and mean standardised uptake value (SUVmax and SUVmean), metabolic tumour volume (MTV), total lesion glycolysis (TLG) and intratumoral heterogeneity index (HI). The previous parameters were recorded both for the primary tumor (-T) and neck lymph nodes (-N). The following endpoints were evaluated: local control (LC), progression-free survival (PFS) and overall survival (OS). The survival analyses were performed using the Kaplan–Meier method. Univariate analysis was performed using the log-rank test.

**Results:**

Patients with a lower HI-T, SUVmax-T, SUVmean-T and TLG-T had significantly better 2-year LC. In predicting PFS, we found that both lower HI-T and HI-N had significantly better prognosis. However, the OS was only statistically associated with HI-T.

**Conclusion:**

^18^ F-FDG based heterogeneity appears to be an potential predicator of patient survival after treatment.

## Background

Nasopharyngeal carcinoma (NPC) is unique in terms of epidemiology, pathogenesis, and physical history compared to the anatomically adjacent cancers of the head and neck area. It is etiologically associated with the Epstein-Barr virus, and shows an endemic distribution: incidence rates are highest in South-Eastern Asia, including Malaysia, Indonesia, Singapore and South-Eastern China, and as such is the sixth most common cancer among males in the region [1].

NPC is a very radiosensitive tumor [2]; in addition, concurrent chemoradiation (CCRT) and induction chemotherapy (Chemo) have been shown to improve tumor control and survival in patients with locally advanced NPC [3,4]. However, some patients may still develop locoregional and distant failure, thus requiring salvage or palliative therapy [5-7]. Although traditional prognostic factors may provide some useful clinical information, they cannot predict treatment outcome reliably. Therefore, substantial research efforts have focused on the identification of novel prognostic factors to further stratify risk groups with the goal of developing individualized treatment strategies for these patients.

As a molecular imaging modality, ^18^ F-FDG PET/CT has been used in patients with NPC for the initial diagnosis and staging workup [8-10]. Several investigators have examined the value of ^18^ F-FDG PET functional parameters, such as maximum standard uptake value (SUVmax) and mean SUV (SUVmean) for predicting the prognosis of NPC [11-13]. Recently, other semi-quantitative parameters, for instance, metabolic tumor volume (MTV) and total lesion glycolysis (TLG) are becoming a topic of interest in cancer research. Some studies showed that MTV and TLG are important independent risk factors in primary NPC patients [14-17]. However, these parameters have not been sufficiently evaluated because they yielded conflicting results.

Intratumoural heterogeneity has been demonstrated to be a strong indicator of malignant transformation, and one might hypothesize that the texture of intratumoural tracer uptake on PET may represent a likewise useful parameter [18]. Therefore, our study was aimed to investigate ^18^ F-FDG parameters, including ^18^ F-FDG based heterogeneity for predicting survival in patients with locally advanced NPC.

## Materials and methods

### Patient Selection

Forty consecutive patients with newly diagnosed (the interval between date of diagnose and FDG scan date was about 1 week), biopsy-proven locally advanced NPC referred to our center between November 2006 and December 2011 for whole-body ^18^ F-FDG PET/CT were retrospectively included. Patients with metastatic disease at presentation (M1 stage), with other malignancies, or who had been previously treated at other institutions were excluded. All patients were staged according to 2010 American Joint Committee on Cancer staging system.

Pretreatment evaluation was composed of whole-body ^18^ F-FDG PET/CT, and routine procedures including complete medical history, physical examination, indirect or fiberoptic endoscopic examination of nasopharynx, biopsy of the neoplasm in nasopharynx, head and neck Magnetic Resonance Imaging (MRI) scans. These examinations were performed within 2 weeks before treatment. Our study need not get an approval from the review board because it was only a retrospective study. However, PET/CT is not covered by insurance in China, and informed written consent was obtained from our patients before examination.

### PET/CT imaging

^18^ F-FDG was produced automatically by cyclotron (Siemens CTI RDS Eclips ST, Knoxville, Tennessee, USA) using Explora FDG_4_ module in our center. Radiochemical purity was over 95%.

Before the ^18^ F-FDG PET/CT, all the patients were requested to fast at least 4 h. At the time of the tracer injection (dosage: 7.4 MBq/kg), the patients presented blood glucose level under 10 mmol/L. Before and after injection, patients were kept lying comfortably in a quiet, dimly lit room. Scanning was initiated 1 h after administration of the tracer. The images were obtained on a Siemens biograph 16HR PET/CT scanner (Knoxville, Tennessee, USA). The transaxial intrinsic spatial resolution was 4.1 mm (full-width at half-maximum) in the center of the field of view. The data acquisition procedure was as follows: CT scanning was first performed, from the proximal thighs to head, with 120 kV, 80 ~ 250 mA, pitch 3.6, rotation time 0.5. Immediately after CT scanning, a PET emission scan that covered the identical transverse field of view was obtained. Acquisition time was 2 ~ 3 min per table position. PET image data sets were reconstructed iteratively by applying the CT data for attenuation correction, and coregistered images were displayed on a workstation.

### Imaging interpretation

A multimodality computer platform (Syngo, Siemens, Knoxville, Tennessee, USA) was used for image review and manipulation. Two experienced nuclear medicine physician evaluated the images independently. The reviewers reached a consensus in cases of discrepancy.

Quantification of glucose metabolic activity was obtained using the SUV normalized to body weight. The SUVmax and SUVmean for primary tumor (SUVmax-T, SUVmean-T) and neck lymph nodes (SUVmax-N, SUVmean-N) were calculated. Besides, glucose metabolic tumor volume (MTV) was also recorded. The boundaries were drawn large enough to include the primary tumor within the nasopharynx or neck lymph nodes in the axial, coronal, and sagittal PET images. To define the contouring margins around the target, we used an SUV of 2.5. The contour around the target lesion inside the boundaries was automatically produced and the voxels presenting SUV intensity of greater than 2.5 within the contouring margin were incorporated to define the MTV. The TLG was calculated according to the following formula: TLG = SUVmean × MTV. A quantitative measure of intratumoral heterogeneity, heterogeneity index (HI) was obtained by dividing SUVmax by SUVmean for primary lesion and nodal disease [18,19].

### Treatment and follow-up

Primary curative treatment consisted of Chemo + radiotherapy (RT) or Chemo + CCRT, according to the tumor stage and other clinical characteristics. As for RT, all patients received Intensity-Modulated Radiation Therapy (IMRT) for a cumulative dose of 70.4Gy in 32 fractions. We used TPF protocol for Chemo, which consisted of docetaxel 75 mg/m^2^ IV on day 1, cisplatin 75 mg/m^2^ IV on day 1, and 5-Fu 500 mg/m^2^/d continuously IV on 1-5 day. With respect to CCRT, cisplatin 40 mg/m^2^ was used IV weekly during radiation. The treatment protocal was approved by the Nasopharygneal carcinoma multidisciplinary team in our hospital after discussion.

Patients were followed every 3 months in the first to second year, then every 6 months in the third to fifth year and once a year thereafter. In each visit, medical history, physical examination and nasopharyngoscopy were performed. Nasopharyngeal MRI was performed 3 months and 1 year after completion of radiotherapy, and every 6 months in the second to fifth year, and then yearly thereafter. The following tests were done at least every year: chest CT or X-ray, abdominal sonography, and bone scan when clinically indicated.

Distant metastasis was proven by clinical and radiologic evidence, and pathologic evidence if possible. For those without pathologic evidence and obvious clinical symptoms, radiologic evidence would play a more important role. If radiologic evidence was not strong enough to prove metastatic disease at primary diagnosis, then a metastatic lesion might be proven by its enlargement or regression during or after chemotherapy.

### Statistical analysis

We identified progression disease (PD) according to RECIST1.1. The following endpoints were evaluated: local control (LC), progression-free survival (PFS) and overall survival (OS). The PFS duration was measured from the date of first scan to the date of disease progression or was censored at the last follow-up date. Survival was defined as the time between the date of diagnosis and the date of death or last follow-up. The optimal SUVmax cut-off value for each endpoint, which showed the best trade-off between sensitivity and specificity, was determined by receiver operating characteristic (ROC) analysis following the method of Metz [20]. The survival analyses were performed using the Kaplan–Meier method. Univariate analysis was performed using the log-rank test. Data was analyzed by SPSS 13.0 software. All analyses were two-sided. A *p* value less than 0.05 was taken to indicate a significant difference.

## Results

### Patient characteristics and treatment outcome

The demographics and clinical characteristics of the patients were showed in Table 1. In our study, the median follow-up period was 30.5 months (range from 24.0- to 68.0 months). At the end of follow-up, among the 40 patients, 35 patients were alive and 5 patients had died, 5 experienced local recurrences and distant metastases happened in 10 patients. Distant metastasis was the main treatment failure, and the common sites of distant failure were liver, lung and bone.

**Table 1:** **Patient demographics and clinical characteristics**

**Demographic or clinical**		**No. of patients**	**%**
**Characteristic**		**(n = 40)**	
Age	Median	52.5	
	Range	28 ~ 70	
Gender	Male	29	72.5
	Female	11	27.5
Histology	Squamous cell carcinoma	8	20.0
	Non-keratinizing carcinoma	32	80.0
T stage	3	25	62.5
	4	15	37.5
N stage	0	4	10.0
	1	10	25.0
	2	17	42.5
	3	9	22.5
Treatment modality	Chemo + RT	21	52.5
	Chemo + CCRT	19	47.5

Chemo = induction chemotherapy, RT = radiotherapy, CCRT = concurrent chemoradiotherapy.

### Univariate analysis

Univariate analysis of prognostic factors for 2-year LC, PFS and OS were summarized in Table 2. The optimal cut-off value of HI-T, SUVmax-T, SUVmean-T, MTV-T, TLG-T, HI-N, SUVmax-N, SUVmean-N, MTV-N and TLG-N, determined by ROC analysis, were 2.9, 15.6, 4.7, 28.9 ml, 249.1 g, 2.3, 7.6, 5.4, 11.1 ml and 80.4 g, respectively.

**Table 2:** **General characteristic and results of the univariate analysis of risk factors associated with LC, PFS and OS**

**Parameters**	**No. of patients**	**LC**		**PFS**		**OS**	
		**2-year rate (%)**	***p***	**2-year rate (%)**	***p***	**2-year rate (%)**	***p***
Age (year)							
<53	20	95.0	0.782	80.0	0.634	95.0	0.776
≥53	20	94.7		70.0		95.0	
Gender							
Male	29	100.0	0.016	72.4	0.868	96.6	0.644
Female	11	81.8		81.8		90.9	
Histology							
Squamous cell carcinoma	8	87.5	0.685	75.0	0.863	87.5	0.212
Non-keratinizing carcinoma	32	96.8		75.0		96.9	
T stage							
3	26	96.2	0.276	73.1	0.592	96.2	0.721
4	14	92.3		78.6		92.9	
N stage							
0-2	31	93.4	0.239	77.4	0.811	93.5	0.734
3	9	100.0		66.7		100.0	
Treatment modality							
Chemo + RT	21	95.0	0.086	76.2	0.594	95.2	0.968
Chemo + CCRT	19	94.7		73.7		94.7	
HI-T							
<2.9	23	100.0	0.028	100.0	<0.0001	100.0	0.022
≥2.9	17	87.8		41.2		88.2	
SUVmax-T							
<15.6	29	100.0	0.001	72.4	0.933	96.6	0.641
≥15.6	11	81.8		81.8		90.9	
SUVmean-T							
<4.7	23	100.0	0.006	69.6	0.929	95.7	0.942
≥4.7	17	88.2		82.4		94.1	
MTV-T (ml)							
<28.9	13	100.0	0.111	76.9	0.684	100.0	0.848
≥28.9	27	92.4		74.1		92.6	
TLG-T (g)							
<249.1	27	100.0	0.006	74.1	0.976	96.3	0.898
≥249.1	13	84.6		76.9		92.3	
HI-N							
<2.3	26	92.3	0.500	84.6	0.009	96.2	0.411
≥2.3	14	100.0		57.1		92.9	
SUVmax-N							
<7.6	20	95.0	0.692	75.0	0.672	95.0	0.891
≥7.6	20	94.7		75.0		95.0	
SUVmean-N							
<5.4	30	93.3	0.232	76.7	0.634	96.7	0.088
≥5.4	10	100.0		70.0		90.0	
MTV-N (ml)							
<11.1	28	92.9	0.125	78.6	0.907	96.4	0.270
≥11.1	12	100.0		66.7		91.7	
TLG-N (g)							
<80.4	32	93.6	0.235	75.0	0.429	93.8	0.770
≥80.4	8	100.0		75.0		100.0	

**Figure 1:**
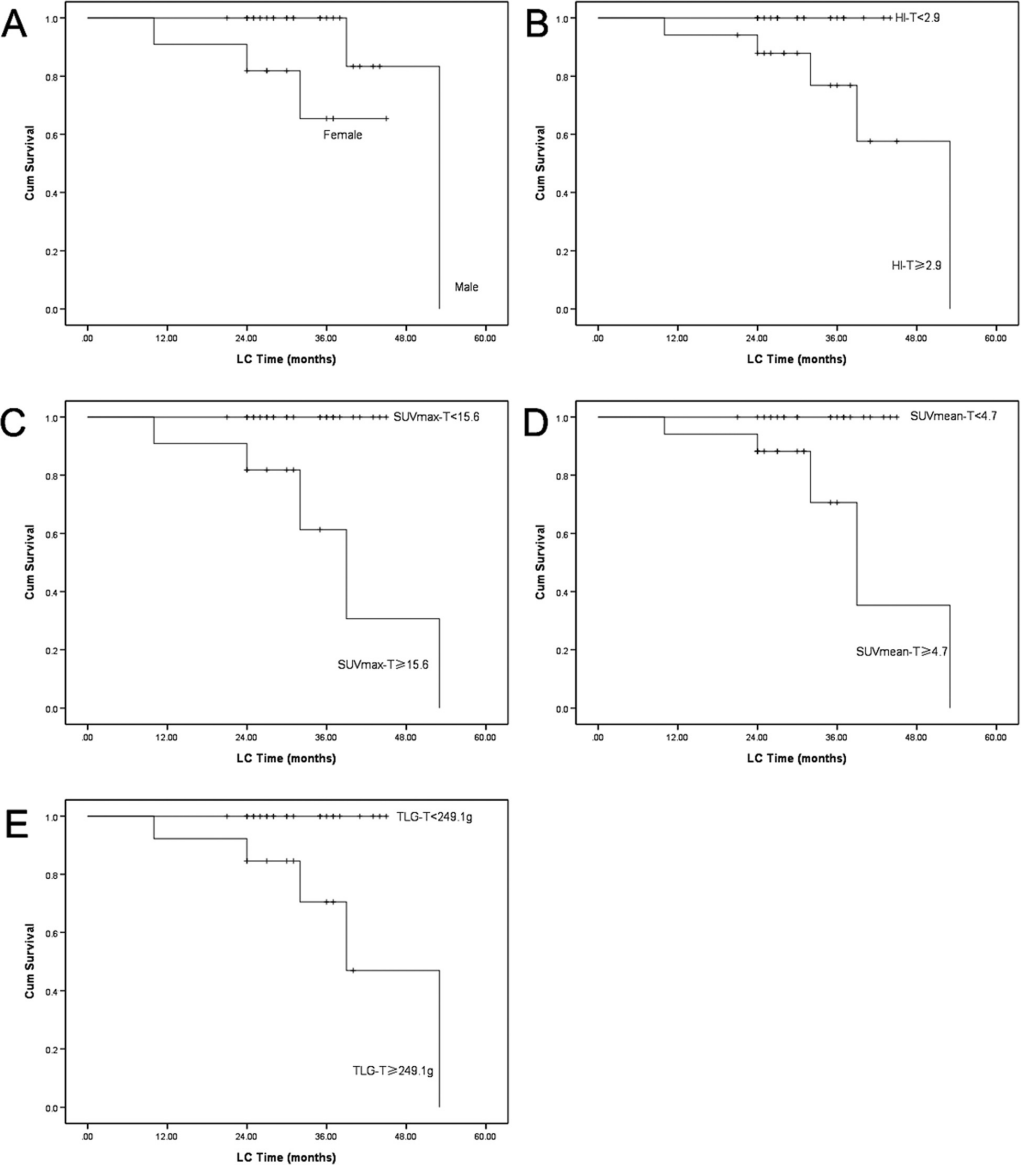
**The Kaplan–Meier curves for the LC of locally advanced NPC patients (A Gender; B HI-T; C SUVmax-T; D SUVmean-T; E TLG-T;** ***p*** **< 0.05).**

**Figure 2:**
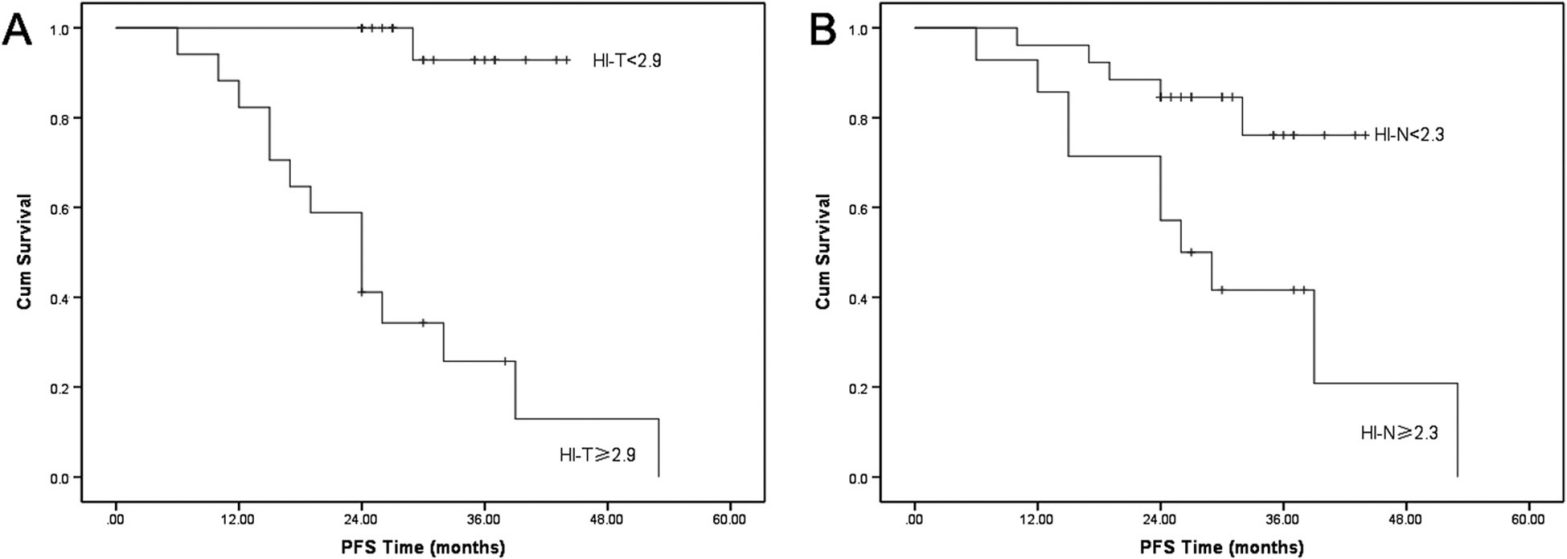
**The Kaplan–Meier curves for the PFS of locally advanced NPC patients (A HI-T;B HI-N;** ***p*** **< 0.05).**

**Figure 3:**
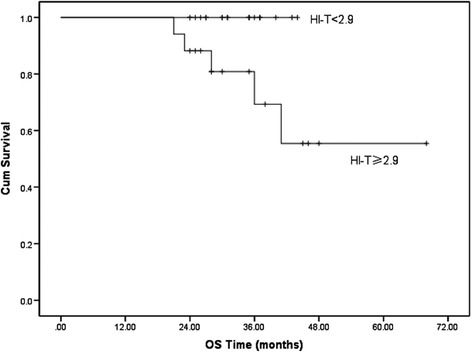
**The Kaplan–Meier curve of different HI-T group for the OS of locally advanced NPC patients (** ***p*** **= 0.022).**

We first examined the significance of traditional prognostic factors in the entire study cohort. The results showed that only gender was significantly associated with LC. With regard to the PET parameters, patients with a lower HI-T, SUVmax-T, SUVmean-T and TLG-T had significantly better 2-year LC (Figure 1). In predicting PFS, we found that both lower HI-T and HI-N had significantly better prognosis (Figure 2). However, the OS was only statistically associated with HI-T (Figure 3).

## Discussion

Prognostic factors for malignant tumors have always been attracting a lot of attention, because such a result could allow therapy to be tailored to the characteristics of an individual patient [21,22]. However, modern cancer care is critically dependent on imaging technologies. As a molecular imaging technology, PET can provide information about the functional or metabolic characteristics of malignancies, tumor stage and therapeutical response, and tumor recurrence; whereas conventional imaging technologies predominantly assess anatomical or morphological features of the tumor including its size, density, shape and so on [23,24]. There have been researches indicating that ^18^ F-FDG PET may non-invasively predict tumor response to treatment and reflect biologic aggressiveness of tumor such as cell viability, proliferative activity, hypoxia, low apoptosis rate, and P53 over-expression in head and neck tumors [25].

Recently, PET-derived parameters, such as the SUV and MTV, have shown potential prognostic value for primary NPC in several reports [11-17]. But most of them only analyzed the predictive value of no more than two PET parameters, and always focused on traditional parameters. Additionally, the results were varied and controversial. Among them, the SUV was the most widely used predicator. Several investigations suggested SUV could be valuable in prognosis [11-14,17] while some studies showed its uselessness [15,16]. Besides, most previous studies failed to point out the definite pattern of treatment failure (for instance, local recurrence or distant metastasis) which PET parameters may predict, so the conclusions were not able to guide which kind of more aggressive treatment, such as additional boost dose or chemotherapy, should be used in patients of high-risk predicted by ^18^ F-FDG PET.

In our study, SUVmax-T and SUVmean-T was associated with LC with a cut-off value of 15.6 and 4.7, which was higher than previous studies (SUVmax ranged from 6.48 to 12.0) [11-14,17]. In view of our enrolled patients, who were all locally advanced NPC, we arbitarily considered that the higher SUVmax was attributed to higer staging (T3-4) with more aggressive potential.

Recent investigators demonstrated that MTV and TLG were better than SUV alone for predicting prognosis[14-17]. Furthemore, TLG would be expected to provide a better prognostic stratification than either MTV or SUV because it integrates both anatomic (tumour volume) and biological data (glucose metabolism) theoretically. Nevertheless, our study showed TLG was only valuable in LC; Therefore, whether TLG was better than SUV or MTV in predicting prognosis of NPC was still unclear.

It is recognized that malignant tumors exhibit intratumoral biological heterogeneity associated with cellular and molecular characteristics such as cellular proliferation, necrosis, fibrosis, differences in blood flow and angiogenesis, cellular metabolism, hypoxia and expression of specific receptors, some of which may be evident on histological analysis. Similarly, heterogeneity of ^18^ F-FDG uptake within tumors has been attributed to a number of factors including cellularity, proliferation, angiogenesis, necrosis and hypoxia, factors that independently have been associated with more aggressive behaviour, poorer response to treatment and worse prognosis [26].

Intratumoral heterogeneity in ^18^ F-FDG PET images has been evaluated using textural analysis [27-29], the coefficient of variance (COV) [30], cumulative SUV-volume histograms (CSH) [31], the area under the CSH (AUC–CSH) [30,32] and fractal analysis [33]. However, the methods mentioned above were too complex to be used in clinical application. Recently, a feasible quantitative measure of heterogeneity, HI, has been successfully applied [18,19]. HI was obtained by dividing SUVmax by SUVmean, which could be easily measured in practice. In our study, we found that both HI-T and HI-N could be potentially used in predicting survivals. The consequences indicated that we should pay more attention to the tumor with greater heterogeneity.

The Radiation Therapy Oncology Group has conducted a study of concurrent chemoradiotherapy followed by adjuvant chemotherapy with bevacizumab (RTOG 0615). The results indicated that the therapy is feasible and may delay the progression of subclinical distant disease [34]. Another phase II clinical trial of cetuximab with concurrent chemoradiotherapy in locoregionally advanced NPC also showed that the strategy is feasible and the preliminary rate was favorable comparing with historic data [35]. It is our opinion that more aggressive systematic treatment may be considered in the patients with high risk of treatment failure, which could be predicted by HI-T of FDG PET.

Of course, the present study had several limitations. The first was the retrospective nature of the study. Another limitation was the relatively small number of patients, which might have led to the selection bias. The third and the largest drawback was the low number of events.

## Conclusion

Our preliminary study demonstrated that pretreatment ^18^ F-FDG based heterogeneity appears to be an potential predicator of locally advanced NPC patient for disease progression and overall survival after treatment. These findings provide additional evidence supporting the use of ^18^ F-FDG PET in the clinical management of patients with NPC.

## Abbreviations

NPC: Nasopharyngeal carcinoma
SUV: Standardised uptake value
MTV: Metabolic tumour volume
TLG: Total lesion glycolysis
HI: Heterogeneity index
CCRT: Concurrent chemoradiation
Chemo: Chemotherapy
MRI: Magnetic Resonance Imaging
RT: Radiotherapy
IMRT: Intensity-Modulated Radiation Therapy
LC: Local control
PFS: Progression-free survival
OS: Overall survival
ROC: Receiver operating characteristic
COV: Coefficient of variance
CSH: Cumulative SUV-volume histograms
AUC–CSH: Area under the CSH
